# Qualitative Study Examining Attendance for Secondary School Pupils With Long-Term Physical Health Conditions

**DOI:** 10.5334/cie.111

**Published:** 2024-05-15

**Authors:** Vicky Hopwood, Simon Pini, Bethan K. C. Spencer, Cath Kitchen

**Affiliations:** 1 University of Leeds, Leeds Institute of Health Sciences, UK; 2 National Association for Hospital Education, UK

**Keywords:** Long-term physical health conditions, children, young people, attendance, absence, school

## Abstract

For some children and young people (CYP) with long-term physical health conditions (LTPHCs) attending school can be difficult. There is a lack of evidence documenting their school attendance experiences, how schools manage absence for these children, and subsequent effects. This study utilised an existing dataset from eighty-nine 11–18-year-olds in mainstream secondary schools in the United Kingdom across 11 LTPHCs that provided first-hand accounts about the children’s experiences of school. Data pre-coded “attendance” were subject to thematic analysis to explore issues emerging. Findings showed attendance patterns varied, with some CYP missing little and others significant amounts of education. Children with LTPHCs wanted to attend school and did their best to navigate education alongside health. School systems for attendance were inconsistent and adversarial. Remedial and supportive action emerged as lacking, and children felt it was their personal responsibility to make up for lost time and missed work when absent. Decisions on whether they attended school, coupled with practices promoting high attendance had detrimental consequences for CYP with LTPHCs educationally, emotionally and socially. Overall, children with LTPHCs felt punished, stigmatised, unfairly treated, unequal and excluded. Results have implications for education, health, and policy practitioners to better plan and target attention so that the LTPHC cohort are treated sensitively and equitably and afforded their entitlement to education when they cannot go to school for health reasons often outside of their control.

## Introduction

School attendance is currently a key challenge for schools. Attendance and absence are long-researched, complex, and multi-disciplinary issues. Attending school is generally linked to a range of benefits for pupils, whilst absence is associated with a range of negative consequences (Kearney et al., 2022). Specifically, school absence has been argued to negatively affect children and young people’s (CYP) academic skills and attainment (Allison et al., 2019; Department for Education, 2016; Hancock et al., 2013) and reduce their opportunities for social and emotional growth (Gottfried, 2014; Heyne & Brouwer-Borghuis, 2022). Being included in the school community creates a “sense of belonging,” which has positive effects on health and well-being (Haim-Litevsky et al., 2023; Pini et al., 2019; Riley et al., 2018). If CYP are not in school, and part of the school community, they can miss consequent health and well-being benefits. Absence from school is linked to increased risks to child welfare, mental health, criminality, depression, violence, and adult unemployment (The Centre for Social Justice 2023; Department for Education, 2022; Heyne & Brouwer-Borghuis, 2022; Kipp, 2022).

### Impetus to Reduce Absenteeism

There is growing pressure on governments, schools, and practitioners to address increases in persistent absenteeism post Covid. In the United Kingdom (UK) this has led to calls for “zero percent” school absence policies and a rallying call to encourage CYP back into school as a matter of “social justice” (The Centre for Social Justice, 2023; Children’s Commissioner, 2022). Consistently attending school is considered a good way of “equalising opportunities” for all children (Benhenda, 2023), particularly because levels of absence disproportionately affect vulnerable groups, including pupils from lower socio-economic backgrounds, ethnic minorities, and those with disabilities (Kearney et al., 2022, 2023).

### Tackling Absenteeism

Globally a plethora of research and policy attention over many years have been directed toward tackling absenteeism to mitigate the negative effects of not being in school. This includes research focussing on interventions to tackle different “types” of absenteeism; for instance, school refusal (where young people openly refuse to go to school as it causes them emotional distress and do not hide absence from their parents) and truancy (where young people deliberately do not attend school without the consent or knowledge of their parents) (Baskerville, 2022; Heyne & Brouwer-Borghuis, 2022).

Studies have variously examined different theoretical frameworks and approaches designed to redress low attendance. These include those positioning absence as a condition within a pupil’s control, those seeking to change pupil behaviour, foster well-being, and promote school sense of belonging, and those seeking to tackle the emotional factors contributing to school absence (Baskerville, 2022; Halligan & Cryer, 2022; Kearney et al., 2022; Kipp, 2022). On-the-ground school practices include using incentives to enhance attendance but with varied levels of success (Balu & Ehrlich, 2018) and punitive measures involving the use of discipline, exclusion, and legal interventions for attendance (Department for Education, 2022). Positive interventions for improving attendance are considered somewhat effective, whilst negative interventions adopting punitive and sanction-based methods can lead to worsening absenteeism (Kearney et al., 2022).

### UK Law and Pupils With LTPHCs

In the UK parents are legally responsible for ensuring their children receive a “fulltime, suitable education” (Education Act, 1996). As such, parents are responsible for ensuring their children routinely attend and can be fined or prosecuted if they do not (Department for Education, 2015a, 2022). Schools, for their part, have a legal duty to take care of children with medical conditions (Bainham & Gilmore, 2015; Department for Education, 2013, 2015b; Children and Families Act, 2014) and acknowledge that these children may face added difficulties to attending school.

Although there is no agreed official data (Bernell & Howard, 2016; Spencer et al., 2022), there are an estimated 1–1.7 million CYP with a LTPHC in the UK (All Parliamentary Group for Diabetes, 2017; Hagell et al., 2013; Lewis & Lenehan, 2014; National Institute for Health and Care Excellence [NIHCE], 2023; Spencer et al., 2022). Using estimates based on available headcount figures for 2022/2023 (Gov UK, 2023) and the estimated figure of 1.7 million CYP in England (NIHCE, 2023), this equates to approximately 11–17% of the pupils in England living with a LTPHC (Gov UK, 2023).

CYP with LTPHCs are shown to have lower rates of attendance in school due to their condition often resulting in symptoms, side-effects and healthcare appointments (Eloi et al., 2019; Jay et al., 2023; Lum et al., 2017; Polderman et al., 2010; Spencer et al., 2023a; Suris et al., 2004). They have also been shown to have lower academic attainment than their healthy peers (Forrest et al., 2013; Knight et al., 2018), although a 2023 review questioned whether school attendance is the meditating factor in the attainment of young people with LTPHCs (Jay et al., 2023). Sense of school belonging is regarded as an important psychological need for CYP with LTPHCs (Tomberli & Ciucci, 2021). As a result, emerging discourse is highlighting the value of collaborating with CYP to better shape the resources and services designed to support them (Lea et al., 2018; Taylor et al., 2018; Van Schelven et al., 2021).

Whilst there is recognition in UK policy that CYP with LTPHCs face additional barriers to attending school, there is scant evidence on how attendance and absenteeism are experienced by and managed for CYP with LTPHCs, along with a dearth of evidence on how practices to promote attendance affect this population. Better documentation and understanding of these issues using first-hand accounts from CYP will contribute to informing existing debates on tackling absenteeism. It will also help to better understand whether practices around attendance for CYP with LTPHCs are in line with equalities legislation and educational rights and guidance (Department for Education, 2022; Education Act, 1996; Equality Act, 2010; Jay et al., 2023).

### The INSCHOOL Project

The INSCHOOL project is 5-year research programme conducted in the UK between 2020 and 2025 to better understand and document the school lives of CYP with LTPHCs. To date, the project has reviewed existing research in this topic area (Spencer et al., 2022) and conducted a large-scale qualitative study with CYP across a diverse range of LTPHCs (Spencer et al., 2023a, 2023b). Using methods co-designed with CYP to collect first-hand accounts of school experiences (Spencer et al., 2023b), the qualitative project highlighted the significant unmet needs of this cohort and substantial commonalities in their school experiences regardless of their specific health condition (Spencer et al., 2023a). Data from the INSCHOOL project provide a source of rich information to explore the school attendance experiences of young people with a LTPHC.

### The Present Study

This paper was based on a secondary analysis of pre-existing qualitative data obtained from the INSCHOOL project. Whilst primary research involves data collection and analysis, this secondary data analysis uses the data already collected for the INSCHOOL project but for another purpose (Carter, 2013).

This new thematic analysis of the pre-existing data aimed to explore issues relating to attendance while leveraging the rich information already gathered on the needs of pupils with LTPHCs (Spencer et al., 2023a). The analysis explored the following research questions:

1. What do secondary pupils with a LTPHC say about their experiences relating to attendance at school?
2. What do their accounts tell us about how attendance is managed and supported at school and about the impact on CYP with LTPHCs?

## Method

### Participant Details

The study sample comprised 89 CYP with a LTPHC (see Table 1) recruited from one of 11 clinics in a Children’s Hospital in the North of England (asthma, allergies, chronic pain, colorectal surgery, cystic fibrosis, dermatology, diabetes, neuromuscular, oncology, rheumatology, and sickle cell anaemia). Sampled CYP ranged in age from 11–18 years and all attended mainstream secondary schools. The sample contained CYP with diversity of gender, age, and ethnicity. Sixty-six of the 89 CYP from the INSCHOOL qualitative sample had transcript extracts coded under the attendance node. Given the freedom to discuss any aspect of their school lives, three quarters specifically chose to raise issues about attendance, demonstrating that it is a notable area of concern for CYP with LTPHCs.

**Table 1:** Participant Characteristics.


Age	11–13	39

	14–15	26

	16–18	24

Gender	Male	41

	Female	48

	Non-binary	0

Ethnicity	White	53

	Asian	22

	Black	11

	Mixed White/Asian	2

	Other	1

Health Condition	Allergies	7

	Asthma	9

	Chronic Pain	8

	Colorectal Surgery	8

	Cystic Fibrosis	10

	Dermatology	9

	Diabetes	10

	Neuromuscular	5

	Oncology	9

	Rheumatology	9

	Sickle Cell Anaemia	5


### Design

A thematic analysis approach was adopted for this study to examine the secondary data. In the initial analysis of the qualitative data gathered for the INSCHOOL project, all information related to attendance was coded under a generic “attendance” node within NVIVO. The present study sought to examine the data coded under the “attendance” node. For detailed descriptions of the data collection methods employed to generate the primary accounts from CYP with LTPHCs, and from which our secondary data analysis was extracted, please see Spencer et al. (2023a, 2023b).

### Analysis

A total of 211 extracts had been broadly coded under the “attendance node” in the original INSCHOOL project but had not been analysed further. In this secondary analysis study, a thematic approach to analysis was employed to construct the common themes present in the attendance experiences of participants (Braun & Clarke, 2006; Kelle 2007; Nowell et al., 2017). The Research Fellow (VH) followed Braun and Clarke’s recommended six stages of thematic analysis. That is, all extracts were examined thoroughly, labelled and coded, before being organised into themes and subthemes that exemplified comparable meanings (Vaismoradi et al., 2016). Emergent themes were then discussed with co-authors SP and BS, who conducted data collection for the INSCHOOL qualitative project, to provide further context and perspective to the emerging themes. To ensure the analysis remained grounded in the experiences and perspectives of CYP, and in line with the participatory approach adopted throughout the INSCHOOL project, a young adviser with lived experience of a health condition independently analysed the attendance data. The young adviser was given an overview of thematic analysis, a broad coding frame, and access to the anonymised interview extracts. The fit of the coding frame along with any concepts and experiences the young adviser saw emerging from the data were then discussed, refined, and agreed to before the thematic framework was finalised.

## Results

Thematic analysis of the attendance node resulted in identification of three themes:

1. CYP absence and factors affecting it
2. School reactions and responses to absence
3. Impacts on CYP with LTPHCs

The themes will be described below accompanied by relevant extracts to exemplify the experiences reported by participants.

### CYP Absence and Factors Affecting It

All participants had missed some time from school. Whilst some reported minimal absence from school, others had missed significant periods of education. Amounts of time taken off school fluctuated based on the extent of need to manage and treat the condition, including short sporadic attendance, repeat absences, or a block of time off over a sustained period.

“I don’t really miss days off, because of my disease.” – 14y Diabetes

“All the days I took off school was equivalent to one academic year.” – 16y Dermatology

Participants were absent from school because they were unwell, experiencing a sudden increase in symptoms or side-effects, undergoing treatment or surgery, or attending medical appointments. Several participants also described missing school because of secondary psychological effects of their condition, such as depression, anxiety, or temporary reduced cognitive functioning. For some, anxiety was increased because of concerns about returning to school after a period of sustained absence. In these cases, participants reported worrying about being subjected to unkind comments from peers because of visible signs of their condition such as rashes, weight changes, scars, hair loss, crutches, or medical devices.

“I’ll have like bumps on my head, so I wouldn’t say I use it as an excuse, but I wouldn’t feel confident enough to go into school.” –14y Dermatology

The participant accounts showed that CYP were “*very orientated around education*” and often made sustained efforts to attend school. Health appointments were scheduled to minimise the amount of time off or to avoid missing core subjects and “*important classes like English [and] maths*.” CYP talked of making compromises between health and education needs when making decisions about attending school, which meant sacrificing one area for the other. Some made determined efforts to carry on with life in the same way they would if they did not have a LTPHC, at times even to the detriment of their health. Alongside their internal motivation to attend school for their own benefit, several felt “*pressured*” to attend school by teachers or parents. Some worried that they would be perceived as if they “*didn’t care”* about education. This meant going to school when they did not feel well enough. Others considered health the main priority and had accepted, or “*resigned*” themselves to, the necessity of time away from school.

“My health comes first. I’ve realised over the years, as long as I’ve tried, even if I go in for an hour, I’ve tried, or some days I’d go in the morning … So, it didn’t matter for me for my attendance because at the end of the day that’s not really important. I did as best as I could.” – 16y Chronic Pain

“Obviously your attendance matters because they [other education providers] want to see how dedicated you are to your education and how motivated you are, so it doesn’t look good, despite me being so dedicated … I feel like my attendance showed I didn’t care and I definitely did.” – 14y Dermatology

Participants’ descriptions showed that they were largely off school for legitimate reasons such as ill health and medical appointments over which they had little control. CYP with LTPHCs wanted to go to school in most cases and were often wrestling with competing health, academic, and psychosocial issues in their decisions and ability to attend.

### School Reactions and Responses to Absence

A few participants reported that their school had been supportive in how they logged and dealt with absence. However, the majority felt their schools’ approach was adversarial with regular and insistent need for justification. Some reported disparity around absence recording with inconsistency in the need for absences to be authorised with examples of schools not authorising an absence depending on the type of medical appointment CYP had.

“It used to be a rule that if you had like doctors’ appointments or something that you couldn’t like schedule yourself, then your attendance wouldn’t go down, but obviously if it was like dentist or something then it would.” – 17y Chronic Pain

Several participants explained they had been challenged on why they were absent and had to provide a defence or agree to change their behaviour. Some had been subjected to home visits where they and their parents were questioned about their attendance. This was rarely described as a supportive process, and participants often felt they were being compelled to attend or not believed. Some had sought support from their doctor to justify reasons for absence.

“I think they was [sic] asking something like ‘why has he had to have so much time off school?’ and then … my doctor had to ring up and tell them.” – 16y Cystic Fibrosis

“They did a home visit and I was not happy, because they know that I’m not being cheeky and doing it on purpose. So that really annoyed me and I got quite upset. I’m at home …, trying to sleep it off so I could be in school, but they didn’t understand that, which really annoyed me.” – 16y Chronic Pain

“They were like trying to make me promise that I’d make my attendance better… I was like, ‘It’s not my fault, like I didn’t choose to have this condition, I didn’t choose my attendance … it’s for hospital appointments.” – 17y Chronic Pain

School practices for dealing with absence were regularly reported as unconstructive, stigmatising CYP with LTPHCs as troublesome. Participants also experienced negative consequences for missing parts of a school day and spoke of being given “*red*” marks when they were late to a lesson because of a medical appointment or late to school due to effects of their condition.

Even when participants were attending, schools adopted different stances on whether they should be included or excluded in certain lessons or permitted to attend school trips. Whilst some participants reported being “*side-lined*” during lessons such as PE, others were required to take part even if advice from their health practitioner had said otherwise.

“PE. In Year 7 I’ve never been to a PE lesson, which is not very good for inclusivity.” – 12y N*euromuscular*

Participants described how their schools deployed incentive and reward schemes for high attendance at school. Many CYP talked about “*missing out*” on these schemes. The practices excluded them, made them feel guilty for not being at school, or caused them to feel they had let their classmates down.

“Every week … we get told our percent and stuff, how much time we’re in school, and people get awards for having good attendance … it’s not so much that I’m being told that my attendance is bad and needs to be improved, but just more people are being congratulated for their good attendance where for me it’s not something that can be helped … I happen to go to hospital sometimes … Then I miss out on awards … that is not a choice … Everyone got like a certificate and a sweet something like that, I just didn’t get one, even though I went to hospital and I came back to school in the snow.” – 11y Cystic Fibrosis

Use of incentives and reward schemes left participants feeling upset, punished, blamed, left out, and unsupported. Participants reported feeling frustrated because they could not control whether they were ill yet were still expected to be present and to contribute to attendance targets. Some participants reported being actively disciplined or cautioned for having low attendance, chastised in class for missing work, or prevented from progressing to the next phase of education.

“What really upsets me is when school has reward days if you’ve got over 97% and you’re there and all the class has gone for pizza and you’re sat in the class and you’re like ‘it’s not my fault’.” – 16y Chronic Pain

“It almost makes the teachers a bit mad at you in a way that you’ve not come into their class or you’ve come in late.” – 16y Chronic Pain

“At one point … I got told like, ‘If your attendance goes so low then you can’t move up into Year 13,’ which me and my mum was like [sic], ‘No, it’s not my fault that I’m going to hospital appointments.’” – 17y Chronic Pain

More supportive approaches to managing attendance reportedly flexed around participants’ health condition. These included authorising absence without question, having participants back into school on phased returns, and not marking them down as late in recognition that their being late was due to their condition or adverse effects of medication.

Participant feedback showed that practical support from schools to catch up and keep up with education missed was variable and unsystematic. Often it was provided by a sympathetic individual or a supportive teacher rather than as a planned school response. There was little communication about the work participants had missed when they were off. This meant they were not aware of lesson coverage or missed notification of upcoming tests. Participants often relied on friends for information. There was little evidence of participants being offered alternative, novel, or compensatory forms of education. Participant data contained limited feedback suggesting use of online approaches, and where these were available, participants did not find them very helpful or effective.

Most participant accounts showed they felt the onus was on them to compensate for time missed and that they were responsible for catching up with work in their own time.

“Sometimes we fall behind in class and you try and catch up.” – 16y Cystic Fibrosis

“It was just a kind of do-it-yourself sort of thing … it was never practical to do and it was never something school really wanted to do.” – 16y Chronic Pain

In sum, participants’ comments showed they felt unsupported. Keeping and catching up required extra personal effort on top of challenging health needs.

### Impacts on CYP With LTPHCs

Academically, participants felt they were not fulfilling their potential, did not have equal access to education as their peers, and were missing out. They felt left behind, and that they were “*not getting the same education as everybody else”*. Being off school meant missing important learning, tests, or exams. Intermittent absence could mean they missed key elements of the curriculum that were built on in subsequent lessons and left participants with gaps in their knowledge. Combined with an unsystematic approach to catching up on missed work, this left participants at a clear disadvantage.

“I had a test and this was like the first time that I had done so badly on a test and I was … upset. I also felt like I shouldn’t feel like I didn’t do it properly, I should kind of understand that I didn’t have as much as an opportunity of getting higher grades as other people because obviously I’d missed out on so much.” – 14y Dermatology

“I feel like I’m being pushed behind … and this isn’t good enough.” – 16y Chronic Pain

Alongside academic difficulties, physical and mental health consequences were experienced. Managing both health and education needs could be exhausting and overwhelming, with many participants describing a challenging cycle of falling behind and catching up that left them feeling physically “*drained*” from trying not to let “*hospital life*” overlap with “*school life*”.

“Sometimes I just get a bit exhausted if I’m having to go to school and I don’t really want to be there.” – 11y Cystic Fibrosis

“At one point it was just a cycle of sleeping, school catching up, sleeping, school catching up, and I think it does get quite like almost dizzy and monotonous because it’s just so continuous.” – 16y Chronic Pain

Returning to school after a diagnosis, a long period of treatment, or surgery was challenging. Participants described this as impacting their mental health, with some having bouts of panic and fear about returning to school after being off.

“I was having panic attacks.” – 16y Dermatology

Participants were also concerned about adverse effects on their parents because of defending their absence from school. Relationships between parents and teachers could suffer when schools compelled parents to get CYP into school when they were not feeling well enough.

“I couldn’t physically get my foot out the door to go to school, even when I was perfectly well, and it was to the point my mum was having to go to school and say, ‘look … there’s really nothing I can do. I don’t know how to help because, you know, she was fine last night, saying she was happy to go and see her friends, but this morning, you know, there’s nothing I can do.’ So it was very much a struggle for my mum to have to manage.” – 16y Dermatology

Missing school impacted negatively relationships and social interaction for participants, with absences making building and maintaining friendship groups challenging. Several described how they felt friends had “*moved on*” while they had been away from school so they were having to start building friendships from “*square one*”.

“It made having friendships difficult, almost to the point of impossible.” – 16y Dermatology

“It was difficult to like engage … with my friends and what they were saying and everything when I’m never there.” – 17y Oncology

Several participants had resigned themselves to the fact that their health required time away from school. They had learned to accept their health condition and had reconciled themselves to how that affected their education.

“If I miss it, it’s fine because it’s just like a day and it’s okay, like it doesn’t really matter.” – 16y Rheumatology

“My attendance, I ain’t bothered, as long as I’m here and healthy and I’m doing my best.” – 16y Chronic Pain

Although there are some positive aspects to this acceptance, participants often described this acceptance in terms of resignation coming from regular experiences of not being actively shown how they could still be included in school and supported to achieve their goals.

## Discussion

The results of our analysis contribute to existing evidence by providing first-hand accounts from CYP with varying physical health conditions on schools’ management of attendance and absence and consequent effects. These accounts offer detail that can be used in support of emerging discourse advocating the need to develop services in collaboration with the young people they aim to support (Lea et al., 2018; Taylor et al., 2018; Van Schelven et al., 2021).

In concordance with the existing research, the data have confirmed that CYP with LTPHCs are consistently missing school, to a greater or lesser extent, for a variety of reasons surrounding their health conditions (Jay et al., 2023; Lum et al., 2017; Polderman et al., 2010; Suris et al., 2004). Adding to current evidence, the participant accounts in the current study provide nuanced detail, showing that CYP with LTPHCs wanted to attend school, made sacrifices to do so, and revealed that attendance is being differentially recorded and monitored. This inconsistent recording and monitoring could result in further inconsistencies in identifying potential unmet needs of CYP and how those needs might be addressed.

Reflecting the literature, absence disadvantaged CYP academically (Allison et al., 2019; Department for Education, 2016; Forrest et al., 2013; Hancock et al., 2013; Knight et al., 2018). Missing time from school meant they missed important aspects of their education and learning. Additionally, participant accounts revealed that support received for education missed was variable and unsystematic. The majority of CYP felt it was their personal responsibility to make up for lost time and missed work, resulting in a draining cycle of falling behind and catching up. This required additional effort from CYP and families, who were already finding managing health alongside school a challenge.

Existing research has suggested that CYP fare better when they feel a “sense of belonging” (Gottfried, 2014; Haim-Litevsky et al., 2023; Heyne & Brouwer-Borghuis, 2022; Riley et al., 2018, 2020). The participant accounts in this study reflected this, showing that when CYP were absent from school, they missed important opportunities for social interaction and friendship. In addition, the data showed that practices encouraging high attendance can adversely affect CYP’s sense of belonging. The emphasis on attendance targets and the use of incentive and rewards systems used in schools often left CYP feeling marginalised, misunderstood, not recognised for the difficulties they were facing, and further excluded from the school community. This finding adds to the evidence showing that in contrast to their healthy peers (Lum et al., 2019), CYP’s sense of well-being at school (Pini et al., 2019) is much lower for those with LTPHCs. It also reflects research on youths who are truant, confirming that CYP need to be “accepted, not belittled; to be trusted, not suspected; to belong, not to be disconnected” (Baskerville, 2022, p. 110). School practices for managing attendance included those that were supportive, recognising that CYP could be absent because of their health condition. Predominantly, however, approaches were deliberately or inadvertently punitive. Far from promoting attendance, these led to a sense of blame or shame increasing psychological or social pressure. These findings lend support to the evidence base showing that punitive actions are ineffective in tackling absenteeism (Kearney et al., 2022; Kipp, 2022).

The findings provide significant rich detail about the complexity of young people’s attendance, adding to the existing discourse positioning absence in different ways. Absence may be considered a choice that CYP control or alternatively as a signal to identify underlying causes from which intervention can be targeted (Halligan & Cryer, 2022; Kipp, 2022). In some cases, our participants chose to attend school despite not feeling well enough, which resulted in worsened physical and mental health. In other cases, they were not able to choose to attend because of the severity of their condition or treatment. That is, their health issues meant they had no control over the situation but could be made to feel punished regardless and left isolated and unsupported. Some participants elected not to go to school due to secondary effects of their health condition such as fear of bullying, stigma, and anxiety, which reflects research on emotionally based school avoidance (Halligan & Cryer, 2022). The data also show that CYP with LTPHCs have unmet needs arising from missing school socially, psychologically, and academically, and that interventions and support to address these were lacking.

Confirming the existing literature, the participant data show that current practices aiming to promote increased attendance or reduce absence to zero can be harmful for this group of CYP and likely ineffective (Jay et al., 2023; Spencer et al., 2023a). Overall, our evidence supports recent research suggesting a need for greater assistance for CYP with LTPHCs and more adaptive attendance policies with resources to keep CYP engaged. Such efforts are needed to enable this group of CYP to exercise their right to education in accordance with equalities legislation (Equality Act, 2010; Jay et al., 2023; Spencer et al., 2023a) and government guidance in England, UK on supporting children with medical conditions in schools (Department for Education 2015b).

## Conclusion

CYP with LTPHCs have inevitable absence from school because of illness and medical appointments. This paper showed that decisions on whether to attend, coupled with school policies and practices to promote high attendance, can have detrimental consequences for this group of students for whom remedial and supportive action is currently lacking. The qualitative data provide a rich source of information directly from children and young people to help policy makers as well as education and health practitioners to better plan and target the attention and support they receive in school to ensure that CYP with LTPHCs are included, treated equitably, and receive their full entitlement to education.

### Limits of the Study

This study was based on direct accounts from CYP using a participant-led approach that prioritises first-hand experience as a valuable source of knowledge (Spencer et al., 2023a). However, the CYP’s views are limited to experiences of mainstream schools from LTPHCs across 11 clinics and, therefore, may not transfer to other educational settings or chronic health conditions. However, there was commonality in the issues raised across CYP from the 11 health clinics included in the study.

As this was a secondary analysis project, attendance was not the sole or primary focus of the data collection. As a result, there may be additional depth to attendance experiences that was not gathered in the main INSCHOOL project.

### Implications for Further Research

There is a need for further research to identify, assess, and monitor the attendance of CYP with LTPHCs and to capture parental and school perspectives. More investigation to categorise and quantify educational, psychological, and social effects of the LTPHC population is required. Specifically, it is important to identify and describe what constitutes effective delivery, quality remedial action, and support around attendance for this cohort. In addition, there is an urgent need to develop protocols for delivery to ensure greater consistency and parity of opportunities across schools.

## Additional File

The additional file for this article can be found as follows:

10.5334/cie.111.s1 Supplementary File 1. Appendix. Sample Quotes Table.
