# Supplementary material for: Qualitative Study Examining Attendance for Secondary School Pupils With Long-Term Physical Health Conditions
Source: Contin Educ. 2024 May 15;5(1):76–89. doi: 10.5334/cie.111 (PMC11104356; doi:10.5334/cie.111)
Supplement: Supplementary File 1. — Appendix. Sample Quotes Table. [file cie-5-1-111-s1.pdf]

Supplementary File 1: Sample Quotes

Theme 1: CYP Absence and Factors Affecting It

**1.1: Patterns of Attendance (attend/not attend)**	
1.1a Don't miss:	*"I don't really miss days off, because of my disease."- 14y Diabetes*<br><br>*"Since I don't take transfusions anymore, I don't miss days of school, the only day I miss school is if I feel sick or very unwell. - 11y Sickle Cell*<br><br>*"Missing like days of school, that would rarely happen."- 12y Neuromuscular*
1.1b Miss:	*"All the days I took off school was equivalent to one academic year." - 16y Dermatology*<br><br>*"I think now, on average, I have like one [appointment] every two weeks but I've started going a lot more now. "I missed quite a few days and I think over the last 7 years I've missed a lot of days, my attendance has been really low some years." - 16y Chronic Pain*<br><br>*"I was never at school."- 17y Oncology*<br><br>*"I missed around about almost 2 years of school." - 16y Oncology*<br><br>*"Having to go to the hospital and have my appointment and then go into school, I was missing half a day at a time, so I was missing loads of lessons." - 18y Chronic Pain*
**1.2: Reasons for non-attendance**	
1.2a Medical appointment	"*You have your hospital appointments. You miss school when you have your hospital appointments." - 16y Chronic Pain*

	"I'd say that hospital appointments, in the beginning, were affecting school as well because I'd have to take a lot of time out of school to have, go and have a blood test or I'd miss lessons because of an appointment." - 16y Rheumatology
1.2b Ill-health – primary & secondary effects of condition	*"When you have a day because you've woken up in absolute agony and going to school isn't the right thing, then it just goes down as red, doesn't it?" - 16y Chronic Pain*<br><br>*"If I'm really tired, then I might have to go in late to school or maybe in extreme cases have the day off just to recover."- 14y Dermatology*<br><br>*"I had to take a whole week off because I had really bad pain in my ankles and I had like blisters and everything, so then I had to take a week off for that, and then I can just have odd days off with like my feet hurting." - 12y Neuromuscular*<br><br>*"I'll have like bumps on my head, so I wouldn't say I use it as an excuse, but I wouldn't feel confident enough to go into school." - 14y Dermatology*<br><br>*"I was having panic attacks; I couldn't physically get my foot out the door to go to school, even when I was perfectly well." - 16y Dermatology*
**1.3: Decision-making factors to attend**	
1.3a Like school/want to or push themselves to go even if ill	"*If I miss days of school, I might miss very important things that I need to do to help me in exams, or if I even miss the exam it's even worse, so I think going to school is very important, it's not good to miss school, but if it's really for a serious reason like you're ill, or you were admitted to the hospital for something like that, you should obviously go and do whatever you have to do, or stay at home to get well. So yeah, going to school is very important to me. – 11y Sickle Cell*<br><br>*"I shouldn't be missing out on things because of this condition I have … it's not as if you and your condition are at war, but it's like you need to understand that it's part of you. So, it's like it shouldn't be a compromisation [sic] but … despite it being there you should be able to do those things that you could have done when it wasn't there." - 14y Dermatology*<br><br>*"I don't miss many, I try not to, I just try to get on with it." - 14y Rheumatology*

	"So I missed quite a bit of school, but I feel like obviously we go to school to learn so I feel like it plays a big part, I really want to prioritise keeping up. It was never as if I had a long period of time off because of it because obviously I understand I need to go back into school, it's really important." - 14y Dermatology<br><br>*"If I feel like I can walk, then I'd definitely go to school, you don't want to miss important classes like English, maths. If it's really bad, we'll probably call the school, tell them that this is happening." - 13y Dermatology*<br><br>*"I don't take time off school because I feel tired, I still go." - 15y Neuromuscular*
1.3b Health issue/Prioritise health	*"I need to stay at home just to get more energy back."- 12y Neuromuscular*<br><br>*"My health comes first. I've realised over the years, as long as I've tried, even if I go in for an hour, I've tried, or some days I'd go in the morning ... So, it didn't matter for me for my attendance because at the end of the day that's not really important. So I did as best as I could in my GCSEs, but the attendance, it was a bit…, but I couldn't help it. So I wasn't really bothered about it at all." - 16y Chronic Pain*
1.3c Don't want to be negatively perceived	*"Obviously your attendance matters because they [other education providers applying to] want to see how dedicated you are to your education and how motivated you are, so it doesn't look good, despite me being so dedicated and so like I'm very orientated around education, I love it, I definitely want to like engage in it and carry on doing it. So I feel like my attendance kind of showed that I didn't care and I definitely did." - 14y Dermatology*<br><br>*"I think they would be a bit like 'oh well this is a bit weird, she's been taking a lot of time off school recently'". - 12y Cystic Fibrosis*<br><br>*"If you miss any school, it goes against your attendance." - 14y Dermatology*

Theme 2: School Reactions and Responses to Absence

**2.1: Approach to absence**	

2.1a Supportive	*"I always get help because they know that sometimes I have to go for appointments because of my condition, so they always help me out, they always cheer me on." - 12y Neuromuscular*<br><br>*"The attendance team, they have like an office and I think it's because I missed so much you just kind of get to know them, and I'll go in there and sometimes I do like say 'I need to go home, like it's getting too much' and they're quite understanding." - 16y Chronic Pain*<br><br>*"They allowed me to come back on a phased return. So I would have to do afternoons because before 12 o'clock I felt like I was going to drop the whole time. ... They've been good and they ... acknowledge the fact that I might not be here for certain days or whatever if I have to go to the hospital or if I just feel you know, not well enough." - 14y Dermatology*<br><br>*"My teacher was just like 'I understand this isn't you to not do as well in the test, like I also understand that you weren't here for as long', so it completely compensates for it and, you know, it's not an excuse but it justifies it." - 14y Dermatology*<br><br>*"Whenever I'm in hospital and have eczema issues, they don't take my attendance off, because they see it's unfair and my disabilities are protected by the government so it's quite, it's all right." - 13y Allergies*<br><br>*"The teachers all know about it, so they'll be reasonable." - 18y Chronic Pain*
2.1b Adversarial<br>Punitive/stigmatising/challenging/missing out	*"It almost makes the teachers a bit mad at you in a way that you've not come into their class or you've come in late." - 16y Chronic Pain*<br><br>*"I think I feel pressured to always come in even if I'm not feeling well, or I'm ill, or something like that, I feel pressure to come into school still. - 11y Cystic Fibrosis*<br><br>*"At one point ... I got told like, 'If your attendance goes so low, then you can't move up into Year 13' ,which me and my mum was like, 'No, it's not my fault that I'm going to hospital appointments.'" - 17y Chronic Pain*

	"I could be quite poorly, and I can't help how that is. I can't stop that from happening obviously. That went together with keeping up with work because I wouldn't be doing the work and then I wouldn't know what I'm doing in the future lessons, but then at the same time I can't help that, that's just because of my CF." - 12y Cystic Fibrosis<br><br>*"It got to a point where I was off too much. They [education] did a home visit once because they didn't believe my mum I was unwell." - 16y Chronic Pain*<br><br>*"What really upsets me is when school has reward days if you've got over 97% and you're there and all the class has gone for pizza and you're sat in the class and you're like 'it's not my fault'." - 16y Chronic Pain*
**2.2: Features of School Approaches**	
2.2a Approach requires justification for time-off	*"I think they was [sic] asking something like 'why has he had to have so much time off school?' and then … my doctor had to ring up and tell them." - 16y Cystic Fibrosis*<br><br>*"They did a home visit and I was not happy, because they know that I'm not being cheeky and doing it on purpose. So that really annoyed me and I got quite upset … they didn't understand that, which really annoyed me." - 16y Chronic Pain*<br><br>*"They were like trying to make me promise that I'd make my attendance better… I was like, 'It's not my fault, like I didn't choose to have this condition, I didn't choose my attendance … it's for hospital appointments.'" - 17y Chronic Pain*
2.2b Differential logging and recording	*"It used to be a rule that if you had like doctors' appointments or something that you couldn't like schedule yourself, then your attendance wouldn't go down but obviously if it was like the dentist or something then it would." 17y - Chronic Pain*
2.2c Approach Includes/excludes including even when present at school	*"PE. In Year 7 I've never been to a PE lesson, which is not very good for inclusivity." - 12y Neuromuscular*<br><br>*"Every week ... we get told our percent and stuff, how much time we're in school, and people get awards for having good attendance … it's not so much that I'm being told that my attendance is bad and needs to*

	be improved, but just more people are being congratulated for their good attendance where for me it's not something that can be helped … I happen to go to hospital sometimes … Then I miss out on awards … that is not a choice … Everyone got like a certificate and a sweet something like that, I just didn't get one, even though I went to hospital and I came back to school in the snow."- 11y Cystic Fibrosis
**2.3: Compensation**	
2.3.a Catch up/keep up	*"I emailed teachers to send me work, like on the days I was off, so I had that. And then some of them also tried to catch me up in lesson, they give me worksheets and stuff to help me out, so that was quite a lot of it. I had some really helpful teachers." - 14y Rheumatology*<br><br>*"It's like they have to [help]… some of them don't bother explaining what's happened [in the lesson missed]." - 16y Chronic Pain*<br><br>*"Sometimes we fall behind in class and you try and catch up." - 16y Cystic Fibrosis*<br><br>*"It was just a kind of do-it-yourself sort of thing … it was never practical to do and it was never something school really wanted to do." - 16y Chronic Pain*<br><br>*"It was just a kind of do-it-yourself sort of thing … because the rest of the time I was just either in the car on the way back to school or on the way to the appointment or in the appointment itself, so it was never practical to do and it was never something school really wanted to do." - 16y Chronic Pain*<br><br>*"I want to get all the knowledge I have to like keep up with the work and like get help from the teachers." - 13y Diabetes*<br><br>*"I always make sure, especially my favourite lessons, that I always catch up on my work, like after my big operation, which was like nearly two years ago, I have a picture of me and I was literally like three days after my operation sat up in my bed doing maths or whatever." - 15y Neuromuscular*

Theme 3: Impacts on CYP with LTPHCs

**3.1: Academic**	

3.1a Reduced academic progress/achievement	*"I feel like I'm being pushed behind … and this isn't good enough." - 16y Chronic Pain*<br><br>*"I missed like 30% of my attendance. So it wasn't great, and then I'd fallen down from Set 1 in maths to Set 2 and my grades weren't as high." - 14y Rheumatology*
3.1b Lack of parity of access to education	*"I had a test, and this was like the first time that I had done so badly on a test and I was … upset. I also felt like I shouldn't feel like I didn't do it properly, I should kind of understand that I didn't have as much as an opportunity of getting higher grades as other people because obviously I'd missed out on so much." - 14y Dermatology*<br><br>*"Obviously, I'm not getting the same education as everybody else." - 16y Dermatology*
3.1c Missing education/learning	*"I feel like I'm being pushed behind a bit and this isn't good enough or I've missed this and I think just keeping up with everything. When you miss days you miss school work, you miss the start, the end of topics, you miss how to do things or you don't know if you're going to have a test or not, and you go back to school, you're just kind of jumping back in and you're like 'I don't know, I don't know what's going on here.'" - 16y Chronic Pain*<br><br>*"I had maths on Monday and I missed it, and then I got set homework, but obviously I didn't know what they did in lesson so I didn't know how to do it. So I was doing this homework and I just didn't know how to do it because I hadn't learnt how to do it, and then I was just confused." - 12y Cystic Fibrosis*
**3.2: Health**	
3.2a Health consequences: Physical health & Mental health	*"Sometimes I just get a bit exhausted if I'm having to go to school and I don't really want to be there." - 11y Cystic Fibrosis*<br><br>*"At one point it were just a cycle of sleeping, school catching up, sleeping, school catching up, and I think it does get quite like almost dizzy and monotonous because it's just so continuous." - 16y Chronic Pain*<br><br>*"I was having panic attacks." - 16y Dermatology*

	"You have your school life and then you have your hospital life and you just get consumed." - 16y Chronic Pain
**3.3: Social**	
3.3a Social consequences	*"It made having friendships difficult, almost to the point of impossible." - 16y Dermatology*<br><br>*"It was difficult to like engage … with my friends and what they were saying and everything when I'm never there." - 17y Oncology*<br><br>*"I really struggled with friends, especially the transition from primary school to high school because I missed loads." - 16y Chronic Pain*
**3.4: Psychological**	
3.4a Psychological/emotional effects	*"I was having panic attacks … I couldn't physically get my foot out the door to go to school, even when I was perfectly well, and it was to the point my mum was having to go to school and say, 'look … there's really nothing I can do. I don't know how to help because, you know, she was fine last night, saying she was happy to go and see her friends, but this morning, you know, there's nothing I can do.' So it was very much a struggle for my mum to have to manage." - 16y Dermatology*<br><br>*"I got quite upset … I'm at home …, trying to sleep it off so I could be in school, but they didn't understand that, which really annoyed me." - 16y Chronic Pain*
**3.5: Acceptance**	
3.5a Acceptance time off required	*"If I miss it, it's fine because it's just like a day and it's okay, like it doesn't really matter." - 16y Rheumatology*<br><br>*"My attendance, I ain't bothered, as long as I'm here and healthy and I'm doing my best." - 16y Chronic Pain*<br><br>*"I'm glad to be back you know and it's made me appreciate what others might not appreciate." - 14y Dermatology*

	"Sometimes you are going to miss a day of school because you were sick, and you can't help being ill, and it's not your fault at all, it's just your body, it's totally normal, your body's going to catch bacteria and you're just going to get ill, you can't cope without helping yourself and just getting rest. If you don't it just, it makes it worse." - 13y Allergies
